# Development and validation of a clinical prediction model for endocervical curettage decision-making in cervical lesions

**DOI:** 10.1186/s12885-021-08523-y

**Published:** 2021-07-13

**Authors:** Yuanxing Li, Haixia Luo, Xiu Zhang, Jingjing Chang, Yueyang Zhao, Jing Li, Dongyan Li, Wei Wang

**Affiliations:** 1 Department of Obstetrics and Gynecology, the Second Hospital of Shanxi Medical University, Taiyuan, 030001 Shanxi China; 2 Shanxi Medical University, Taiyuan, 030000 Shanxi China

**Keywords:** Cervical cancer, Endocervical curettage, Preoperative factors, Nomogram, Decision-making

## Abstract

**Background:**

In the absence of practical and reliable predictors for whether the endocervical curettage (ECC) procedure should be performed, decisions regarding patient selection are usually based on the colposcopists’ clinical judgment instead of evidence. We aimed to develop and validate a practical prediction model that uses available information to reliably estimate the need to perform ECC in patients suspected of having cervical lesions.

**Methods:**

In this retrospective study, 2088 patients who underwent colposcopy, colposcopically directed biopsy (CDB) and ECC procedures between September 2019 and September 2020 at the Second Hospital of Shanxi Medical University were included. The data were analyzed with univariate and multivariable logistic regression. Least absolute shrinkage and selection operator (LASSO) was used to select predictors for ECC positivity. The ECC prediction model was presented as a nomogram and evaluated in terms of discrimination and calibration. Furthermore, this model was validated internally with cross-validation and bootstrapping.

**Results:**

Significant trends were found for ECC positivity with increasing age (*P* = 0.001), menopause (*P* = 0.003), Human papillomavirus (HPV) status (*P* < 0.001), severity of ThinPrep Cytological Test (TCT) (*P* < 0.001), original squamous epithelium ectopia (*P* = 0.037) and colposcopy impression (*P* < 0.001) by multivariable logistic regression analysis. The ECC prediction model was developed based on the following predictors: age, menopause, symptom of contact bleeding, severity of TCT, HPV status, cervix visibility, original squamous epithelium ectopia, acetowhite changes and colposcopic impression. This model had satisfactory calibration and good discrimination, with an area under the receiver operator characteristic curve (AUC) of 0.869 (95% confidence interval 0.849 to 0.889).

**Conclusions:**

A readily applicable clinical prediction model was constructed to reliably estimate the probability of ECC positivity in patients suspicious of having cervical lesions, which may help clinicians make decisions regarding the ECC procedure and possibly prevent adverse effects.

**Supplementary Information:**

The online version contains supplementary material available at 10.1186/s12885-021-08523-y.

## Background

Cervical cancer is the fourth most common cancer among women worldwide, with an estimated 604,127 new cases and 341,831 deaths in 2020 [1]. The detection of high-grade cervical intraepithelial neoplasia (CIN) irrefutably is of interest as a precursor to invasive cervical cancer [2]. At present, the international community follows a three-step screening method: cervical thin-layer liquid-based cytology or cytology combined with human papillomavirus (HPV) detection is performed as the primary screening [3]; cases that are suspicious or positive are referred to colposcopy; and histopathological specimens of the lesions, including colposcopically directed biopsy (CDB) and endocervical curettage (ECC) then might be taken under colposcopy to diagnose cervical lesions [4]. Obtaining the endocervical status of a lesion is a true difficulty for colposcopists. Serious lesions, including cervical cancer, could potentially be missed if ECC is not performed [5]. ECC has been included as a component of clinicians’ armamentarium in the evaluation of women who have abnormal cervical test results by the American Society for Colposcopy and Cervical Pathology (ASCCP) [6]. However, not every woman would benefit from the ECC procedure equally, and there is a long-standing debate over the effectiveness of this procedure, but until now, its value has remained unclear. The additional benefit of ECC in detecting CIN has been found to vary considerably. Moreover, ECC is hard to perform in patients with a stenotic cervix or in menopausal women [7]. The inter-observer agreement of the interpretation of ECC specimens is poor [8, 9]. ECC is an uncomfortable procedure and has been rated as a 5.8 (0 to 10) on a visual analog scale by patients in prior studies [10, 11]. Additionally, the specific indications for ECC remain debated. Colposcopists usually perform ECC selectively based on personal experience instead of the evidence, possibly resulting in a higher incidence of adverse effects [12]. Further identification of subgroups of women most likely to benefit from ECC is urgently needed, but studies have lacked appropriate data or statistical power for such comparisons.

To improve population health outcomes and healthcare costs for patients with CIN or cervical cancer, we need to provide the right treatment to the right people at the right time. In the case of colposcopy, the questions are whether ECC needs to be performed and in which patients. Predicting the probability of positive ECC can be challenging given the considerable heterogeneity in the characteristics of affected individuals and their clinical course as well as the variability in the region of lesions. Reliance on clinical intuition alone might be insufficient for accurate decision-making. This creates the need for a prediction model that aims to assist clinicians in the ECC decision-making process. We focused on a group of patients with ECC results and attempted to construct a visualized model to predict the probability of ECC positivity. Nomograms could provide predictive information tailored to individual patients by establishing a simple, reliable and pragmatic graphical representation of a complex statistical prediction model [13]. Thus, the objective of our study was to develop and validate a clinical prediction model to select patients who would more likely benefit from ECC based on our clinical data.

## Methods

### Study design, setting and population

In this retrospective study, we included all consecutive patients who underwent ECC procedures performed with colposcopy and CDB and were referred to the Second Hospital of Shanxi Medical University, China, based on abnormal co-test findings (HPV testing+cytology) or the presence of unexplained contact bleeding between September 2019 and September 2020. This study was approved by the Ethics Committees of the Second Hospital of Shanxi Medical University. Patients with an abnormal co-test result were defined as those with positive HPV testing results and/or cytology findings demonstrating atypical squamous cells of undetermined significance (ASC-US) and above. Patients were excluded if they had a history of cervical physical therapy (ablation or cryosurgery), surgical operations (loop electrosurgical excision procedure, cold knife cone or hysterectomy) or pelvic radiotherapy, had nondiagnostic or unsatisfactory sampling, or had incomplete charts.

All patients underwent HPV testing, cytology, colposcopy, CDB and ECC procedures. Demographic information, including age, menopausal age and contact bleeding, was obtained via electronic medical records. The cytology technique adopted in this study was the ThinPrep (ThinPrep Pap test, Hologic, MA) Cytological Test (TCT). Cytological results were reported according to the 2014 Bethesda System [14]. The cytological classifications were negative for intraepithelial lesions or malignancy (NILM), ASC-US, low-grade squamous intraepithelial lesion (LSIL), atypical squamous cells cannot exclude an HSIL (ASC-H), high-grade squamous intraepithelial lesion (HSIL), squamous cell carcinoma (SCC), atypical glandular cells not otherwise specified (AGC-NOS), atypical glandular cells-favor neoplasia (AGC-FN), adenocarcinoma in situ (AIS), and adenocarcinoma of the cervix (AC). The HPV testing assays were carried out with flow-through hybridization and a HybriMax gene chip (Hybribio Biotechnology Ltd., China) with the residual Pap test specimens. Twenty-one HPV genotypes can be identified using this assay, including 15 high-risk HPV (HR-HPV) genotypes (HPV16, 18, 31,33, 35, 39, 45, 51, 52, 53, 56, 58, 59, 66 and 68) and 6 low-risk HPV (LR-HPV) genotypes (HPV6, 11, 42, 43, 44 and CP8304 (81)). All of the cytology and HPV tests were performed in the hospitals according to the procedures provided by the suppliers.

Patients with positive HPV testing results and/or abnormal cytology (≥ASC-US) or the symptom of unexplained contact bleeding were referred to colposcopy according to the protocol of this study. Electronic colposcopy (SLC-2000, Goldway, China) was used to visually assess the cervix, including cervix visibility, normal colposcopic findings (columnar epithelium ectopy and transformation zone (TZ) type), acetowhite changes (none, thin or dense) and Lugol staining (stained or nonstained) [15]. The impression of colposcopy includes normal/benign, low-grade, high-grade and cancer [6]. CDB was performed at the squamous-columnar junction in cases of visible lesions. If the colposcopic examination showed no lesions, random 4-quadrant punch biopsies were taken. ECC was performed with a Kevorkian curette. All colposcopy, CDB and ECC procedures were performed by the same gynecologist. Histological diagnoses were graded as NILM, CIN1–3, SCC, AIS or AC by two gynecological pathologists blinded to the cytology findings, HPV testing results and colposcopic impression, based on the Lower Anogenital Squamous Terminology Standardization Project for HPV-Associated Lesions (LAST) [16] and the WHO Classification of Tumors of Female Reproductive Organs (4th edition) [17]. The worst pathological result of CDB and ECC was taken as the final diagnosis.

### Variables selection

All known risk factors for ECC positive from keynote papers and consultation with clinical experts were considered in developing the prediction model. Based on the results of the literature review, we identified the following predictor variables that are assessable before ECC procedure and are consistently associated with our outcome of the prediction model: age [8, 18–23], abnormal cytology [8, 18, 20–22, 24], HPV infection (positive/negative) [20–22], cervix visibility (adequate/inadequate) [18, 22, 24], TZ type (I/II/III) [22] and colposcopic impression [8, 18, 21, 23, 24]. Given the models will be used in general practice, we also add menopause (yes/no), symptom of contact bleeding (yes/no), cervical artrophy (yes/no), original columnar epithelium ectopy (yes/no), acetowhite changes (none, thin or dense), Lugol staining (stained/nonstained) into the prediction model after initial analysis. Meanwhile, we have classified HPV infection in more details, including HPV negative, HPV16 +, HPV18 +, HPV16 and 18 +, HR-HPV+ (non 16/18 types), LR-HPV+. The coding of these variables is outlined in Additional file 1: Table S1.

### Outcome definition

ECC was classified as negative (NILM) or positive (ASC, CIN1–3, SCC, AIS or AC). The outcome of our prediction model was based on all patients with positive ECC findings according to pathology. The diagnosis based on ECC was determined by two gynecological pathologists blinded to the cytology findings, HPV testing results and colposcopy impression based on the criteria of LAST [16]. If the diagnosis or grade based on ECC was not clear during the review, the case was presented to a third pathologist expert blinded to the patient’s potential predictors.

### Sample size

Currently, there is no standard way to calculate sample sizes for prediction models. With 14 predictors for our model, the number of events per variable is over 37. This is sufficient for the development of stable models.

### Model derivation

Baseline characteristics were summarized as frequency tables to study the distributions of categorical variables and as medians and interquartile ranges for continuous variables. Restricted cubic splines were used to explore nonlinearity in the effect of continuous variables. We performed univariable analysis using the chi-square test for each potential predictor to detect any important differences in proportions, with χ2 values and *P* values for categorical variables. Logistic regression was used to estimate multivariable regression coefficients, and the prediction strength was quantified as odds ratios (ORs) with 95% confidence intervals (CIs).

Given the large number of predictors, we used least absolute shrinkage and selection operator (LASSO) regression [25] to reduce the number of candidate predictors and to select the most significant ECC-positive predictors to build the final prediction model. To control the shrinkage procedure, 10-fold cross-validation was used to determine the penalty parameter λ and to develop the optimal model.

### Model performance

The performance of the prediction model was studied in terms of the model calibration (the ability to produce unbiased estimates of the outcome probability) and the model discrimination (the ability to determine whether the patients should undergo ECC). Model calibration was evaluated graphically with calibration plots (plots of predicted versus observed outcomes) [26]. Model discrimination was evaluated with the sensitivity and specificity of the ECC patient selection prediction model analyzed by the receiver operator characteristic (ROC) curve and quantified based on the area under the ROC curve (AUC; equivalent to the c-statistic). The AUC is reasonable above 0.7 and strong above 0.8.

### Model validation

Internal validation evaluates the stability of a prediction model when random changes in sample composition occur. First, 10-fold cross-validation was performed. The model was fitted on a randomly selected subset of 90% of the study patients and tested on the remaining 10% of patients. This process was repeated 10 times to estimate the extent to which the predictive accuracy of the model was overoptimistic. Second, we performed 100 bootstrap resampling, drawn with replacement from the development sample. The model was refitted in each bootstrap replicate and tested to quantify the optimism (the decrease between the performance in the bootstrap sample and the performance in the original sample) in model performance.

### Model presentation

The final model with the shrunken regression coefficients was presented as a regression equation and converted into a nomogram to facilitate clinical application. Each predictor value in the nomogram corresponds to a regression weight such that the total score is equivalent to the linear predictor. For this model, logistic transformation was applied to the linear predictor to produce estimates of the probability of ECC positivity.

### Statistics

All statistical tests were two-sided, and a *P*-value < 0.05 was considered statistically significant. All statistical analyses were performed in the R Studio statistics program (version 1.3.1093), and the glmnet and rms packages in the R Project (version R 4.0.3 GUI 1.73 Catalina build) were used.

## Results

### Patient characteristics

**Fig. 1:**
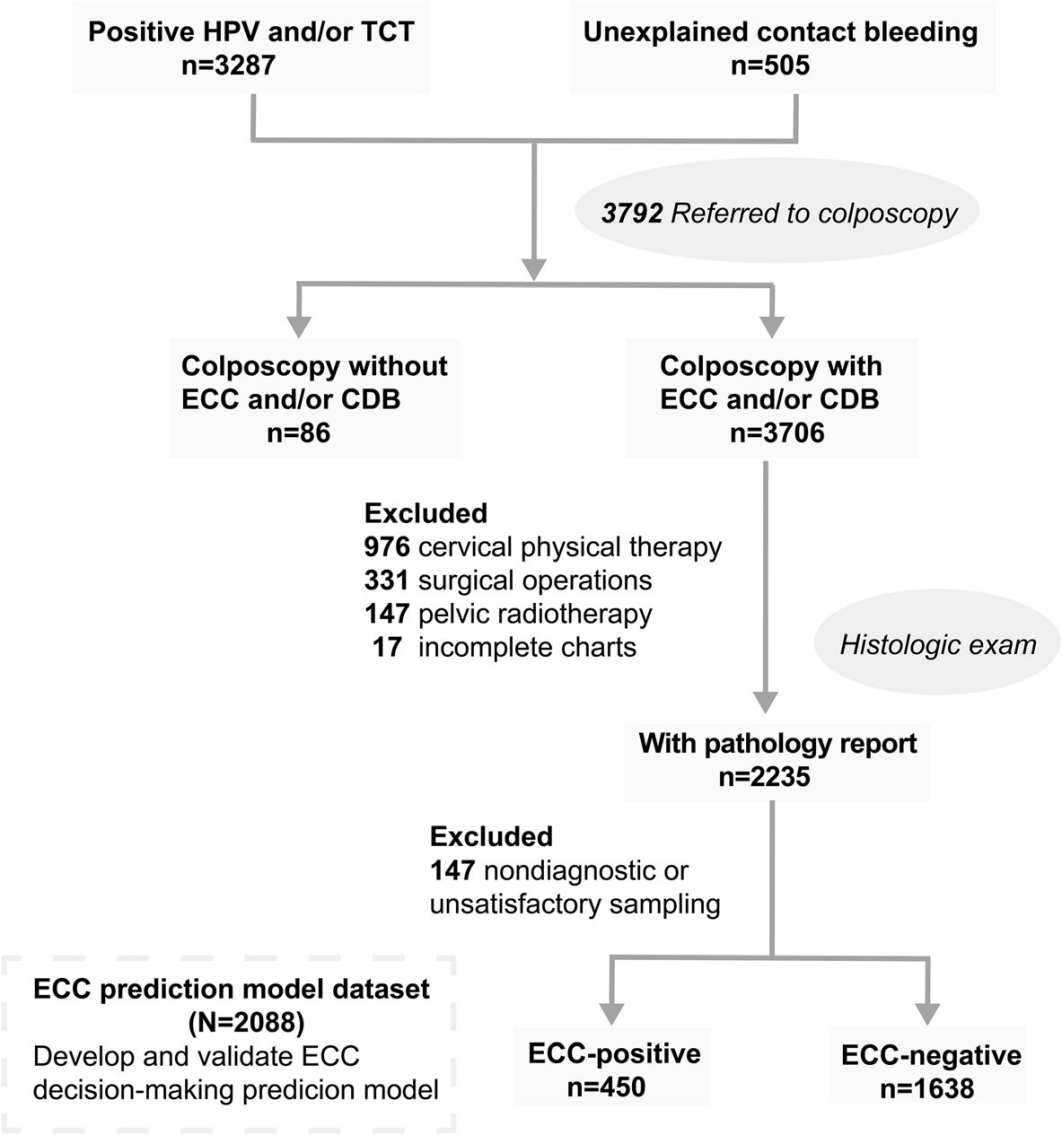
Flow chart for the participants

A total of 3792 patients were referred to colposcopy based on abnormal co-test findings (HPV testing/TCT) or the presence of unexplained contact bleeding. Then 3706 patients underwent ECC and CDB were enrolled. Of these, 1471 patients were excluded based on exclusion criteria, and 147 patients were excluded because of nondiagnostic or unsatisfactory sampling. Two thousand eighty-eight samples were finally included, with 1638 ECC-negative samples and 450 ECC-positive samples (Fig. 1). There were 994 (47.6%) samples with consistent ECC and CDB pathological findings, and 51 (2.4%) samples with a worse diagnosis based on ECC than on CDB. Table 1 compares the distribution of variables between the ECC-positive group and the ECC-negative group. The median age of the patients was 52 (interquartile range 46–60) years in the ECC-positive group and 46 (interquartile range 38–54) years in the ECC-negative group. In univariate analysis, we found that the effect of age could be adequately modeled as a linear function (*P*-nonlinear = 0.6587) (Additional file 5: Figure S1A-B).

**Table 1:** Baseline of clinicopathologic characteristics (*N* = 2088)

Characteristics	ECC positive (*N* = 450)	ECC negative (*N* = 1638)	*χ*^*2*^	*P*-value
Age^a^	52 (45, 60)	46 (38, 54)		
Age groups			132.498	< 0.001
~ 30	3 (0.7%)	104 (6.3%)		
30 ~ 39	54 (12%)	389 (23.7%)		
40 ~ 49	117 (26%)	549 (34%)		
50 ~ 59	152 (33.7%)	425 (26%)		
60~	124 (27.6%)	171 (10%)		
Menopause			97.411	< 0.001
No	172 (38%)	1050 (64%)		
Yes	278 (62%)	588 (36%)		
Symptom of contact bleeding			180.108	< 0.001
No	282 (63%)	1461 (89%)		
Yes	168 (37%)	177 (11%)		
HPV status			426.073	< 0.001
HPV negative	32 (7.1%)	197 (12%)		
HPV16+	264 (59%)	462 (28.2%)		
HPV18+	23 (5.1%)	145 (8.9%)		
HPV16 and 18+	7 (1.5%)	14 (0.9%)		
HR-HPV+ (non 16/18 types)	120 (27%)	790 (48.2%)		
LR-HPV+	4 (0.8%)	30 (1.8%)		
TCT			983.804	< 0.001
NILM	119 (26%)	1000 (61%)		
ASC-US	120 (27%)	379 (23%)		
LSIL	52 (12%)	175 (11%)		
ASC-H	40 (8.9%)	34 (2.1%)		
HSIL	96 (21%)	45 (2.7%)		
SCC	20 (4.4%)	2 (0.1%)		
AGC-NOS	1 (0.2%)	3 (0.2%)		
AGC-FN	1 (0.2%)	0 (0%)		
AIS	0 (0%)	0 (0%)		
AC	1 (0.2%)	0 (0%)		
Cervix visibility			28.407	< 0.001
Inadequate	9 (2.0%)	0 (0%)		
Adequate	441 (98%)	1638 (100%)		
Original squamous epithelium ectopia			23.972	< 0.001
No	414 (92%)	1353 (83%)		
Yes	36 (8.0%)	285 (17%)		
Cervical artrophy			18.697	< 0.001
No	350 (78%)	1411 (86%)		
Yes	100 (22%)	227 (14%)		
TZ type			112.127	< 0.001
Unrecognizable	16 (3.6%)	2 (0.1%)		
Type I	11 (2.4%)	122 (7.4%)		
Type II	309 (69%)	1320 (81%)		
Type III	114 (25%)	194 (12%)		
Acetowhite changes			407.653	< 0.001
None	49 (11%)	514 (31%)		
Thin	112 (25%)	851 (52%)		
Dense	289 (64%)	273 (17%)		
Lugol staining			20.578	< 0.001
Nonstained	443 (98%)	1518 (92.7%)		
Stained	7 (1.6%)	120 (7.3%)		
Colposcopic impression			676.089	< 0.001
Normal/benign	50 (11%)	628 (38.3%)		
Low-grade	99 (22%)	739 (45.1%)		
High-grade	137 (30.4%)	253 (15.4%)		
Cancer	164 (36.4%)	18 (1.1%)		

^a^Statitics presented: Median (IQR); n (%)

We found that there were statistically significant differences between the ECC-positive group and the ECC-negative group in age groups, menopause, symptom of contact bleeding, HPV Status, severity of TCT, cervix visibility, original squamous epithelium ectopia, cervical atrophy, TZ type, acetowhite changes, Lugol staining and colposcopic impression (*P* ≤ 0.001).

**Fig. 2:**
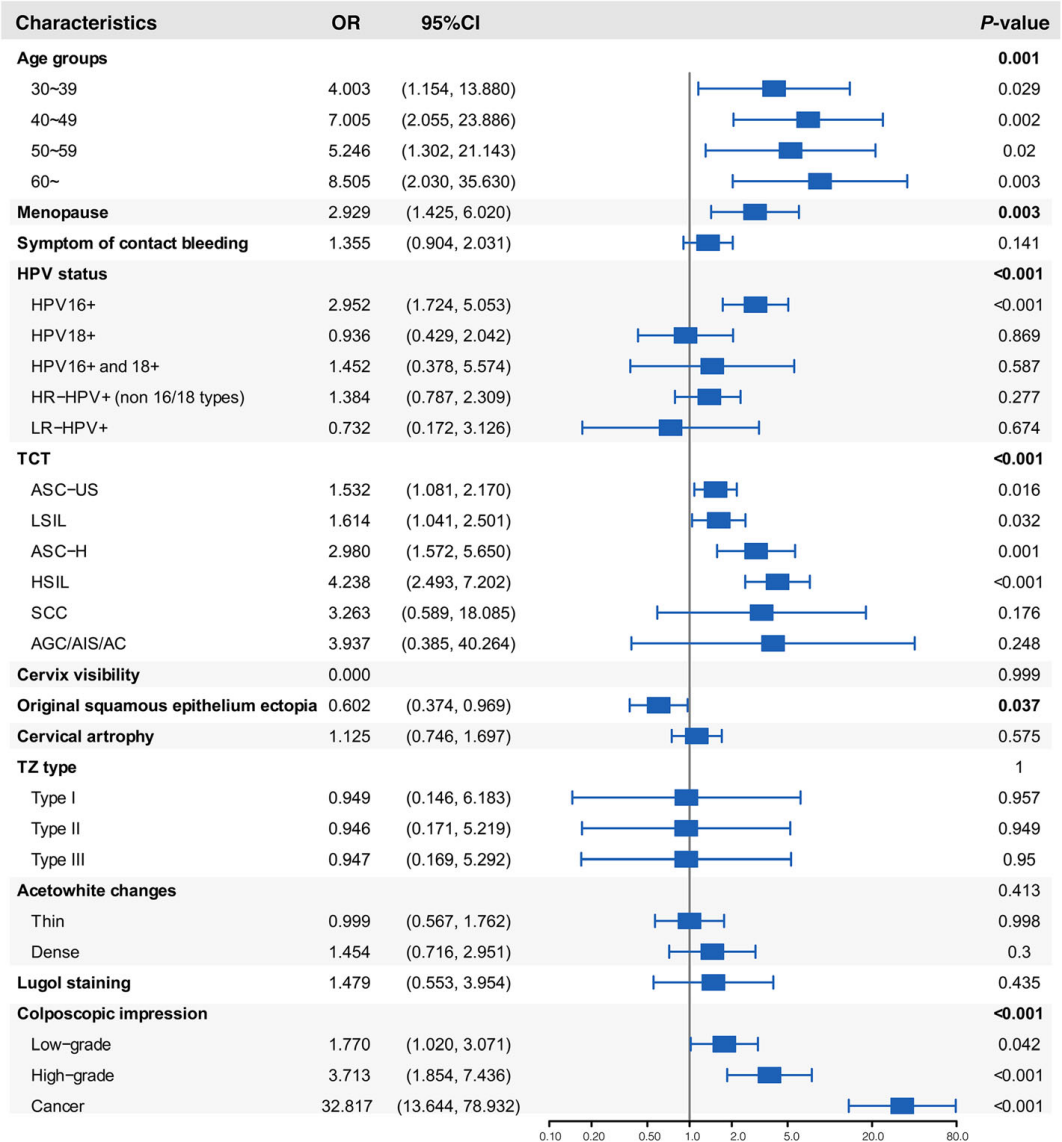
Forest plots of multivariable logistic regression analysis for factors and ECC positivity

Significant trends were found for ECC positivity with increasing age (*P* = 0.001), menopause (*P* = 0.003), HPV status (*P* < 0.001), severity of TCT (*P* < 0.001), original squamous epithelium ectopia (*P* = 0.037) and colposcopy impression (*P* < 0.001) by multivariable logistic regression analysis (Fig. 2 and Additional file 2: Table S2). The risk of ECC positivity in women older than 60 years was 8.505 (95% CI: 2.030, 35.630) times higher than that in women younger than 30 years. Compared with patients with negative cytological findings, those with ASC-US, LSIL, ASC-H, HSIL respectively had 1.532 (95% CI: 1.081, 2.170), 1.614 (95% CI: 1.041, 2.501), 2.980 (95% CI: 1.572, 5.650) and 4.238 (95%CI: 2.493, 7.202) times higher risks of ECC positivity. Compared with patients with a colposcopic impression of normal/benign, those with low-grade, high-grade and cancer had 1.770 (95% CI: 1.020, 3.071) 3.713 (95% CI: 1.854, 7.436) and 32.837 (95% CI: 13.644, 78.932) times higher risks of ECC positivity respectively.

### Predictor selection and model derivation

All other candidate predictor variables were included in the full model. We selected predictors using LASSO. Additional file 6: Figure S2 and Additional file 3: Table S3 show the results of the candidate variables included in the LASSO regression and their corresponding coefficients for the different values of the penalty parameter λ. We observed that when lambda.min was 0.003411379, 11 predictor variables remained. By increasing the λ value for further shrinkage, when lambda.1se was 0.01669495, 9 predictors remained in the final model. The final model included the variables age, menopause, symptom of contact bleeding, HPV status, TCT, cervix visibility, original squamous epithelium ectopia, acetowhite changes and colposcopic impression (Additional file 4: Table S4).

**Fig. 3:**
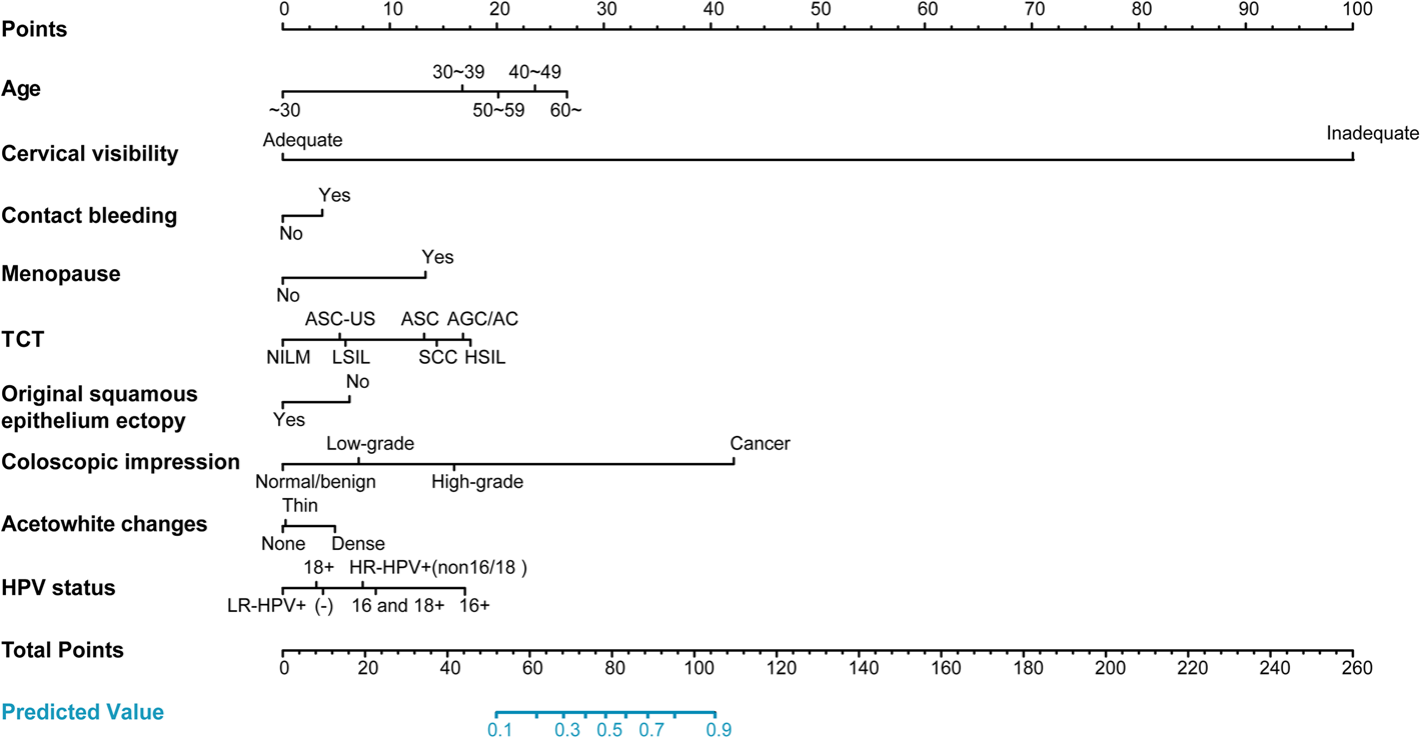
Nomogram predicting ECC positivity

Figure 3 shows the nomogram for predicting the probability of ECC positivity in patients referred for colposcopy. Each predictor is assigned a specific grading value. When the grading value of the 9 predictors is determined, the total score can be obtained by adding them together and the probability of ECC positivity can be calculated.

### Model performance and validation

**Fig. 4:**
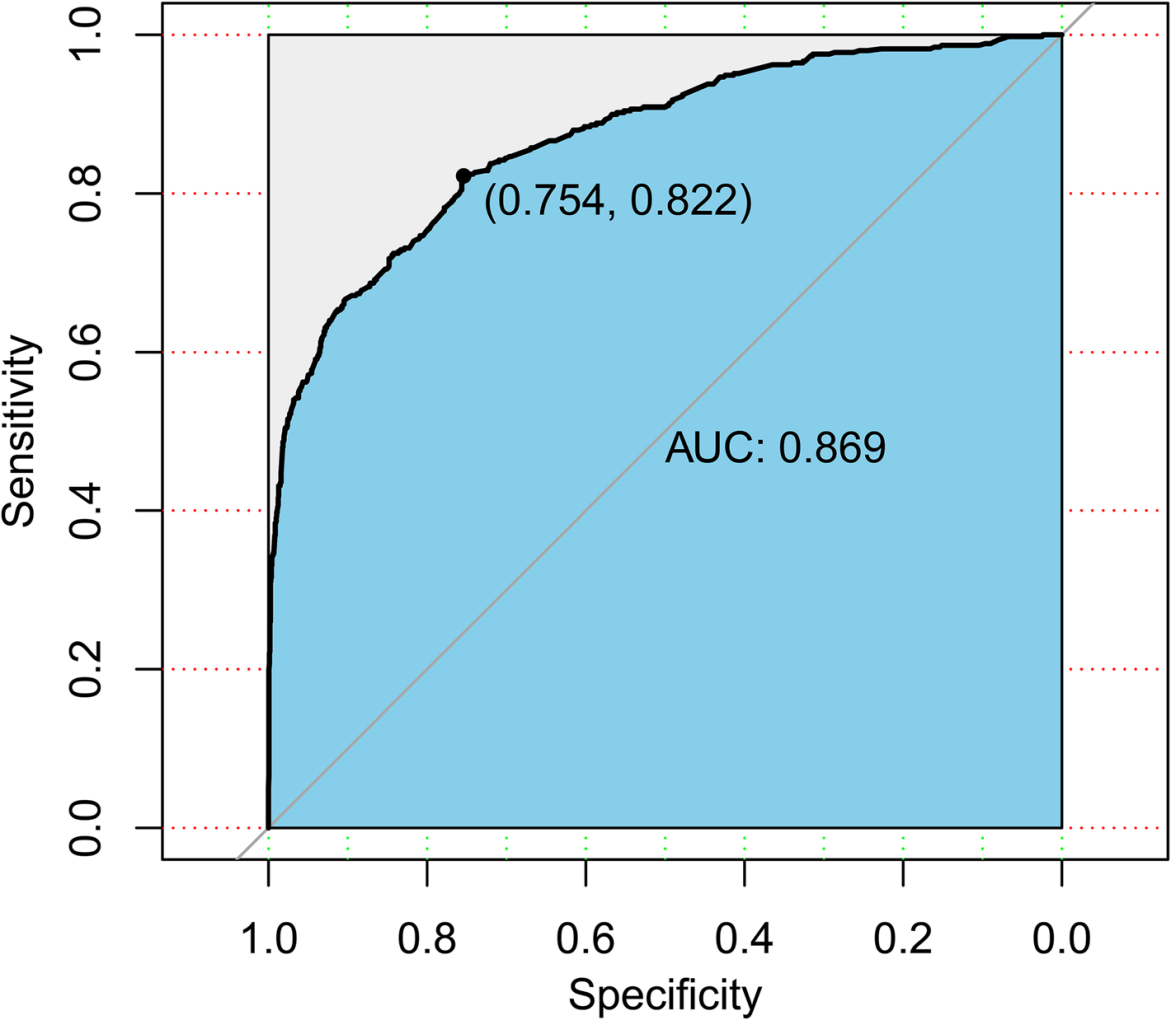
The ROC curve of the nomogram in internal validation

**Fig. 5:**
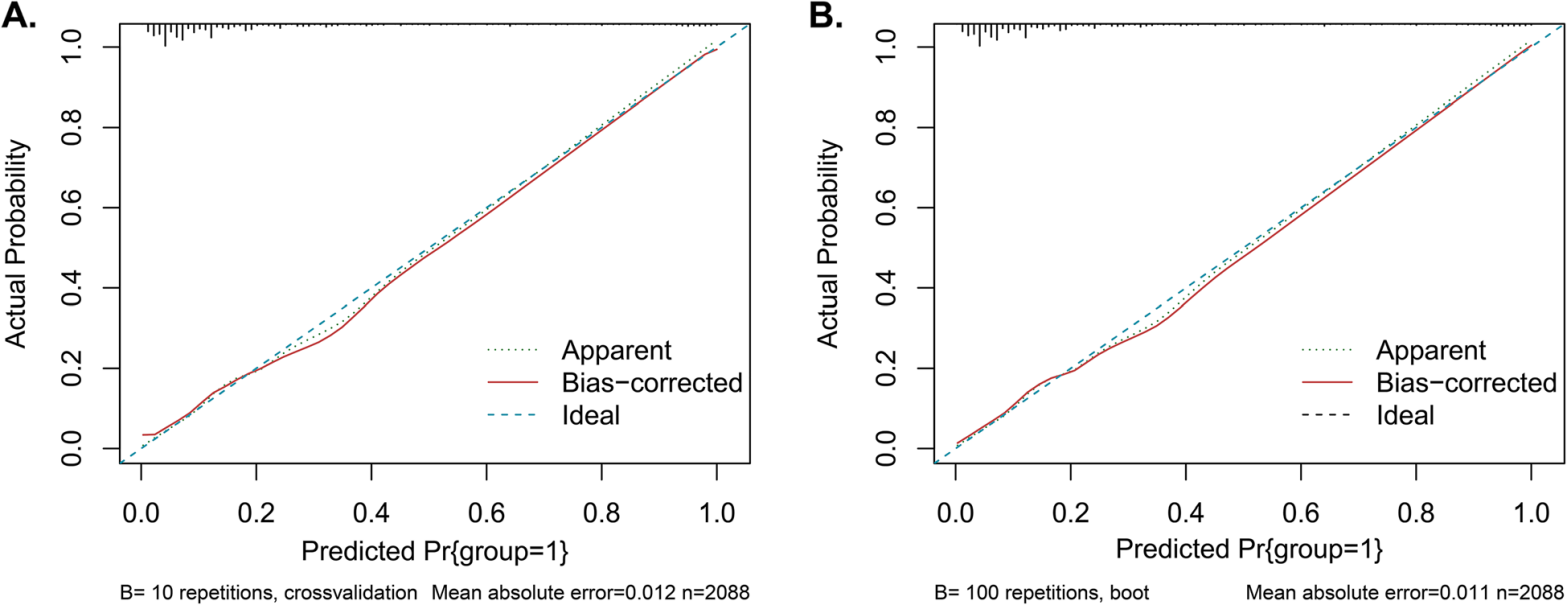
Calibration plots of the predicted and actual probabilities. (**A**) Calibration plots of the predicted and actual probabilities by cross-validation; (**B**) Calibration plots of the predicted and actual probabilities by bootstrapping

The ROC curve of the nomogram in internal validation is shown in Fig. 4. The sensitivity and specificity were 82.2 and 75.4% respectively. The AUC was 0.869 (95%CI: 0.849–0.889). Overfitting was assessed by cross-validation and bootstrapping, and found to be negligible. The final model had an original c-statistic of 0.869 and a modified c-statistic of 0.863 according to 10-fold cross-validation. The apparent c-statistic was 0.859 with 0.0203 optimism according to the bootstrap procedure. Model optimism was negligible, both in the 10-fold cross-validation procedure and with bootstrapping. Calibration plots of the predicted and actual probabilities by cross-validation and bootstrapping were drawn (Fig. 5a-b). The predicted value was basically consistent with the actual ECC positivity rate.

## Discussion

In this study, we screened out and evaluated influencing factors related to ECC positivity using mathematical methods. We proposed a novel ECC patient selection model to assess the risk of ECC positivity in patients referred for colposcopy. Using this model, patients with a higher risk of ECC positivity can be distinguished from those with a lower risk. Our ECC prediction model performed robustly in validation. The model was shown to be well calibrated and have good discrimination. The internally validated AUC was 0.869 (95% CI: 0.849–0.889) for the model to predict the probability of ECC positivity. The predictor items are assessable at hospital admission. The established nomogram could facilitate the adoption of the model into clinical practice.

### Comparison with previous studies

Generally, the yield on ECC seems to be at an increased rate in the setting of inadequate or unsatisfactory colposcopy; in this situation, there is likely less controversy regarding the performance of ECC. In addition to the clear contraindication in certain populations, such as adolescents, immunocompromised patients, and pregnant women, there is also wide agreement that older women, in whom the cervical squamous-columnar junction is more difficult to visualize entirely [27], would benefit more from ECC; however, debate remains over the age cutoff to perform the ECC procedure [8, 18–23].

Another consideration is the grade of dysplasia. ECC was positive in more patients with severe dysplasia than in patients with mild or moderate dysplasia. It has also been reported that a positive ECC finding is directly related to the severity of the cytology results [8, 18, 20–22, 24] and colposcopy impression [8, 18, 21, 23, 24]. Some clinicians base the indication for ECC on the cytologic interpretation or colposcopic impression, although this is not described in guidelines. However, the usefulness of ECC in the setting of high-grade dysplasia as a separate diagnostic tool is debatable. Our results confirm and extend the results from those studies, and our ECC prediction model included cytologic interpretation and colposcopic impression as important predictors.

The evidence for identifying women most likely to benefit from endocervical sampling based on TZ type has also been inconclusive. Research has shown that even when the TZ is completely visible under colposcopy, 5% of patients still have positive ECC findings [22]. Even if the sensitivity of ECC is not strong, it may prompt colposcopists to perform the ECC procedure. Contrary to the findings of most previous studies, our study showed that the TZ type was not an independent predictor of ECC positivity. Incomplete visualization of the TZ is likely more linked to the colposcopist’s experience and may dose not entirely raise concern for missed endocervical lesions.

Notably, we also discovered 5 powerful new predictors: the symptom of contact bleeding, HPV status, cervix visibility, original squamous epithelium ectopia and acetowhite changes. Although the most common reason for the referral of women for colposcopy is abnormal cervical cytology, there are the other indications highly recommendable for colposcopy, such as HPV16/18 positivity, persistent HR-HPV infection and the symptom of contact bleeding. In addition, a study claimed that women ≥30 years with contact bleeding should be referred for colposcopy to rule out the possibility of cervical cancer [28]. Therefore, the results of HPV testing and the presence of contact bleeding are valuable factors to determine whether ECC should be performed. HPV infection, especially HPV 16/18 subtype infection, should particularly raise concern for persistent oncogenic infection and trigger ECC. Interestingly, we subgrouped HPV status into several groups, including HPV negative, HPV16 +, HPV18 +, HPV16 and 18 +, HR-HPV+ (non 16/18 types), LR-HPV+, and HPV16 + was confirmed to be a significant predictor of ECC positive. Changes in the cervix can be highlighted with the application of exogenous contrast agents such as acetic acid and Lugol’s iodine. Moreover, prospective evaluation of the features of the cervix (original squamous epithelium mature/atrophic and columnar epithelium ectopy) was also considered in this analysis. Therefore, to our knowledge, this study is the first to assess the above factors and include them in a prediction model.

With the established model, which incorporates these and other variables, the probability of ECC positivity in women referred to colposcopy can be predicted with a high degree of accuracy.

### Strengths and weaknesses of the study

A strength of the present study is that it was the first to develop and internally validate a model to determine whether the ECC procedure should be performed in cervical lesion screening. Second, to the best of our knowledge, no studies have yet examined the association of HR-HPV genotypes and colposcopic features, and our findings from this study attempted to fill this current knowledge gap by using multivariable logistic regression analysis. Third, LASSO analysis is a method that improves the accuracy of predictor selection and the validation of the model. Nevertheless, there are several potential limitations to our study. First, the model was derived from a retrospective analysis. Another limitation is that the prediction model has not been externally validated. Finally, the treating clinician’s perception of the ECC procedure may have influenced the decision to provide aggressive support for these patients. As a result, the model may partly reflect the clinician’s behavior in treating these patients. This model should be validated in a larger unselected population and re-evaluated in a multicenter study.

### Implications for patient selection, clinical practice, and research

Our findings have implications for the management of colposcopy procedures. It is not known whether patients referred to colposcopy should undergo only CDB or should be aggressively managed with ECC. The selected predictors allowed the development of a highly accurate model for predicting whether ECC should be performed, and the use of an ECC selection nomogram may aid in clinicians’ empirical decision-making for such patients, thus reducing in the overuse of ECC.

## Conclusions

A readily applicable clinical prediction model was constructed to reliably estimate the probability of ECC positivity in patients suspicious of having cervical lesions, which may help clinicians make decisions regarding the ECC procedure and possibly prevent adverse effects.

## Supplementary Information

**Additional file 1: Table S1.** The coding of variables.

**Additional file 2: Table S2.** Multivariable logistic regression analysis of factors associated with ECC positivity.

**Additional file 3: Table S3.** The results of the candidate variables included in the LASSO regression and their corresponding coefficients for the different values of the penalty parameter λ.

**Additional file 4: Table S4.** The including predictors of final ECC prediction model.

**Additional file 5: Figure S1.** Age distribution of model development data and the association of age and ECC positivity (A-B).

**Additional file 6: Figure S2.** The progress of LASSO regression selecting the candidate variables and the penalty parameter λ.

## Data Availability

The data used in the current study are available from the corresponding author upon reasonable request.

## Abbreviations

ECC: The endocervical curettage
LASSO: Least absolute shrinkage and selection operator
HPV: Human papillomavirus
TCT: ThinPrep Cytological Test
AUC: Area under the receiver operator characteristic curve
CI: Confidence interval
CIN: Cervical intraepithelial neoplasia
CDB: Colposcopically directed biopsy
ASCCP: American Society for Colposcopy and Cervical Pathology
ASC-US: Atypical squamous cells of undetermined significance
NILM: Negative for intraepithelial lesions or malignancy
LSIL: Low-grade squamous intraepithelial lesion
ASC-H: Atypical squamous cells cannot exclude an HSIL
HSIL: High-grade squamous intraepithelial lesion
SCC: Squamous cell carcinoma
AGC-NOS: Atypical glandular cells not otherwise specified
AGC-FN: Atypical glandular cells-favor neoplasia
AIS: Adenocarcinoma in situ
AC: Adenocarcinoma of the cervix
HR-HPV: High-risk HPV
LR-HPV: Low-risk HPV
TZ: Transformation zone
LAST: Lower Anogenital Squamous Terminology Standardization Project for HPV-Associated Lesions
OR: Odds ratio
ROC: Receiver operator characteristic
