# Supplementary material for: Development and validation of a clinical prediction model for endocervical curettage decision-making in cervical lesions
Source: BMC Cancer. 2021 Jul 13;21:804. doi: 10.1186/s12885-021-08523-y (PMC8276473; doi:10.1186/s12885-021-08523-y)
Supplement: Supplementary file 1 — Additional file 1: Table S1. The coding of variables. [file 12885_2021_8523_MOESM1_ESM.docx]

**Supplementary Table 1** The coding of variables.

| Variable | Categorical/Binary/Continuous | Coding |
| --- | --- | --- |
| Age groups | Categorical | 1=~30  2=30~39  3=40~49  4=50~59  5=60~ |
| Menopause (yes/no) | Binary | 0=No  1= Yes |
| Symptom of contact bleeding (yes/no) | Binary | 0=No  1= Yes |
| HPV status | Categorical | 0=HPV negative  1=HPV16 +  2=HPV18 +  3=HPV16 and 18 +  4=HR-HPV+ (non 16/18 types)  5=LR-HPV+ |
| TCT | Categorical | 0=NILM／inflammation  1=ASC-US  2=LSIL  3=ASC-H  4=HSIL  5=Squamous cell carcinoma  (SCC)  6=AGC-NOS  7=AGC-FN  8=AIS  9=AC |
| Cervix visibility | Binary | 0=Adequate  1=Inadequate |
| Original squamous epithelium ectopy (yes/no) | Binary | 0=No  1= Yes |
| Cervical atrophy（yes/no） | Binary | 0=No  1= Yes |
| TZ type | Categorical | 0=Unrecognizable  1=Type I  2=Type II  3=Type III |
| Acetowhite changes | Categorical | 0=None  1=Thin  2=Dense |
| Lugol staining | Binary | 0=Stained  1=Nonstained |
| Colposcopic impression  (higest grade) | Categorical | 0=Normal/benign  1=Low-grade  2=High-grade  3=Cancer |
| Histology | Categorical | 0=Inflammation  1=CIN1  2=CIN2  3=CIN3  4=Squamous cell carcinoma (SCC) |
|  |  | 5=Adenocarcinoma in situ (AIS)  6=Adenocarcinoma (AC) |
