# Supplementary material for: Development and validation of a clinical prediction model for endocervical curettage decision-making in cervical lesions
Source: BMC Cancer. 2021 Jul 13;21:804. doi: 10.1186/s12885-021-08523-y (PMC8276473; doi:10.1186/s12885-021-08523-y)
Supplement: Supplementary file 2 — Additional file 2: Table S2. Multivariable logistic regression analysis of factors associated with ECC positivity. [file 12885_2021_8523_MOESM2_ESM.docx]

**Supplementary Table 2** Multivariable logistic regression analysis of factors associated with ECC positivity.

| Characteristics | OR | 95%CI | *P*-value |
| --- | --- | --- | --- |
| Age groups |  |  | 0.001 |
| 30~39 | 4.003 | (1.154, 13.880) | 0.029 |
| 40~49 | 7.005 | (2.055, 23.886) | 0.002 |
| 50~59 | 5.246 | (1.302, 21.143) | 0.020 |
| 60~ | 8.505 | (2.030, 35.630) | 0.003 |
| Menopause | 2.929 | (1.425, 6.020) | 0.003 |
| Symptom of contact bleeding | 1.355 | (0.904, 2.031) | 0.141 |
| HPV status |  |  | <0.001 |
| HPV16+ | 2.952 | (1.724, 5.053) | <0.001 |
| HPV18+ | 0.936 | (0.429, 2.042) | 0.869 |
| HPV16+ and 18+ | 1.452 | (0.378, 5.574) | 0.587 |
| HR-HPV+ (non 16/18 types) | 1.384 | (0.787, 2.309) | 0.277 |
| LR-HPV+ | 0.732 | (0.172, 3.126) | 0.674 |
| TCT |  |  | <0.001 |
| ASC-US | 1.532 | (1.081, 2.170) | 0.016 |
| LSIL | 1.614 | (1.041, 2.501) | 0.032 |
| ASC-H | 2.980 | (1.572, 5.650) | 0.001 |
| HSIL | 4.238 | (2.493, 7.202) | <0.001 |
| SCC | 3.263 | (0.589, 18.085) | 0.176 |
| AGC/AIS/AC | 3.937 | (0.385, 40.264) | 0.248 |
| Cervix visibility | 0.000 |  | 0.999 |
| Original squamous epithelium ectopia | 0.602 | (0.374, 0.969) | 0.037 |
| Cervical artrophy | 1.125 | (0.746, 1.697) | 0.575 |
| TZ type |  |  | 1.000 |
| Type I | 0.949 | (0.146, 6.183) | 0.957 |
| Type II | 0.946 | (0.171, 5.219) | 0.949 |
| Type III | 0.947 | (0.169, 5.292) | 0.950 |
| Acetowhite changes |  |  | 0.413 |
| Thin | 0.999 | (0.567, 1.762) | 0.998 |
| Dense | 1.454 | (0.716, 2.951) | 0.300 |
| Lugol staining | 1.479 | (0.553, 3.954) | 0.435 |
| Colposcopic impression |  |  | <0.001 |
| Low-grade | 1.770 | (1.020, 3.071) | 0.042 |
| High-grade | 3.713 | (1.854, 7.436) | <0.001 |
| Cancer | 32.817 | (13.644, 78.932) | <0.001 |
