# Supplementary material for: Development and validation of a clinical prediction model for endocervical curettage decision-making in cervical lesions
Source: BMC Cancer. 2021 Jul 13;21:804. doi: 10.1186/s12885-021-08523-y (PMC8276473; doi:10.1186/s12885-021-08523-y)
Supplement: Supplementary file 3 — Additional file 3: Table S3. The results of the candidate variables included in the LASSO regression and their corresponding coefficients for the different values of the penalty parameter λ. [file 12885_2021_8523_MOESM3_ESM.docx]

**Supplementary Table 3** The results of the candidate variables included in the LASSO regression and their corresponding coefficients for the different values of the penalty parameter λ.

| Predictor | lambda.min= 0.003411379 | lambda.1se=0.01669495 |
| --- | --- | --- |
| (Intercept) | -1.68816313 | -2.65034882 |
| Age groups |  |  |
| 30~39 |  | -0.03318707 |
| 40~49 | 0.4330304 |  |
| 50~59 |  |  |
| 60~ | 0.47337288 | 0.33887942 |
| Menopause | 1.21893056 | 0.77133993 |
| Symptom of contact  bleeding | 0.2144503 | 0.02923017 |
| HPV status |  |  |
| HPV16+ | 0.79206741 | 0.59927601 |
| HPV18+ | -0.11229971 |  |
| HPV16 and 18+ |  |  |
| HR-HPV+ (non 16/18 types) |  |  |
| LR-HPV+ | -0.15273126 |  |
| TCT |  |  |
| ASC-US | 0.26544799 | 0.02923017 |
| LSIL | 0.31387778 |  |
| ASC-H | 0.90508069 |  |
| HSIL | 1.26818979 | 0.42765027 |
| SCC | 0.72373955 | 0.87141509 |
| AGC/AIS/AC | 0.66291379 |  |
| Cervix visibility | -2.2102001 | -0.0519189 |
| Original squamous epithelium ectopia | -0.44914958 |  |
| Cervical artrophy | 0.09285009 |  |
| TZ type |  |  |
| Type I |  |  |
| Type II |  |  |
| Type III |  |  |
| Acetowhite changes |  |  |
| Thin |  |  |
| Dense | 0.47161892 | 0.80597064 |
| Lugol staining | 0.25261233 |  |
| Coloscopic impression |  |  |
| Low-grade | 0.38166414 |  |
| High-grade | 1.06105587 | 0.35469336 |
| Cancer | 3.22297379 | 2.51002895 |
