# Supplementary material for: Development and validation of a clinical prediction model for endocervical curettage decision-making in cervical lesions
Source: BMC Cancer. 2021 Jul 13;21:804. doi: 10.1186/s12885-021-08523-y (PMC8276473; doi:10.1186/s12885-021-08523-y)
Supplement: Supplementary file 4 — Additional file 4: Table S4. The including predictors of final ECC prediction model. [file 12885_2021_8523_MOESM4_ESM.docx]

**Supplementary Table 4** The including predictors of final ECC prediction model.

| Characteristics | OR | 95% CI | *P*-value |  |  |
| --- | --- | --- | --- | --- | --- |
| Age groups |  |  |  |  |  |
| ~30 | — | — |  |  |  |
| 30~39 | 4.003 | (1.154, 13.880) | 0.029 |  |  |
| 40~49 | 7.005 | (2.055, 23.886) | 0.002 |  |  |
| 50~59 | 5.246 | (1.302, 21.143) | 0.020 |  |  |
| 60~ | 8.505 | (2.030, 35.630) | 0.003 |  |  |
| Menopause |  |  |  |  |  |
| No | — | — |  |  |  |
| Yes | 2.929 | (1.425, 6.020) | 0.003 |  |  |
| Symptom of contact bleeding |  |  |  |  |  |
| No | — | — |  |  |  |
| Yes | 1.355 | (0.904, 2.031) | 0.141 |  |  |
| HPV status |  |  |  |  |  |
| HPV negative | — | — |  |  |  |
| HPV16+ | 2.952 | (1.724, 5.053) | <0.001 |  |  |
| HPV18+ | 0.936 | (0.429, 2.042) | 0.869 |  |  |
| HPV16 and 18+ | 1.452 | (0.378, 5.574) | 0.587 |  |  |
| HR-HPV+ (non 16/18 types) | 1.384 | (0.787, 2.309) | 0.277 |  |  |
| LR-HPV+ | 0.732 | (0.172, 3.126) | 0.674 |  |  |
| TCT |  |  |  |  |  |
| NILM | — | — |  |  |  |
| ASC | 1.532 | (1.081, 2.170) | 0.016 |  |  |
| LSIL | 1.614 | (1.041, 2.501) | 0.032 |  |  |
| HSIL | 2.980 | (1.572, 5.650) | 0.001 |  |  |
| SCC | 4.238 | (2.493, 7.202) | <0.001 |  |  |
| AGC/AIS/AC | 3.263 | (0.589, 18.085) | 0.176 |  |  |
| Cervix visibility |  |  |  |  |  |
| Adequate | — | — |  |  |  |
| Inadequate | 0 |  | 0.999 |  | 0.999 |
| Original squamous epithelium ectopia |  |  |  |  |  |
| No | — | — |  |  |  |
| Yes | 0.602 | (0.374, 0.969) | 0.037 |  |  |
| Acetowhite changes |  |  |  |  |  |
| None | — | — |  |  |  |
| Thin | 0.999 | (0.567, 1.762) | 0.998 |  |  |
| Dense | 1.454 | (0.716, 2.951) | 0.300 |  |  |
| Colposcopic impression |  |  |  |  |  |
| Normal/benign | — | — |  |  |  |
| Low-grade | 1.770 | (1.020, 3.071) | 0.042 |  |  |
| High-grade | 3.713 | (1.854, 7.436) | <0.001 |  |  |
| Cancer | 32.817 | (13.644, 78.932) | <0.001 |  |  |
