# Supplementary figures and images for: Development and validation of a clinical prediction model for endocervical curettage decision-making in cervical lesions
Source: BMC Cancer. 2021 Jul 13;21:804. doi: 10.1186/s12885-021-08523-y (PMC8276473; doi:10.1186/s12885-021-08523-y)

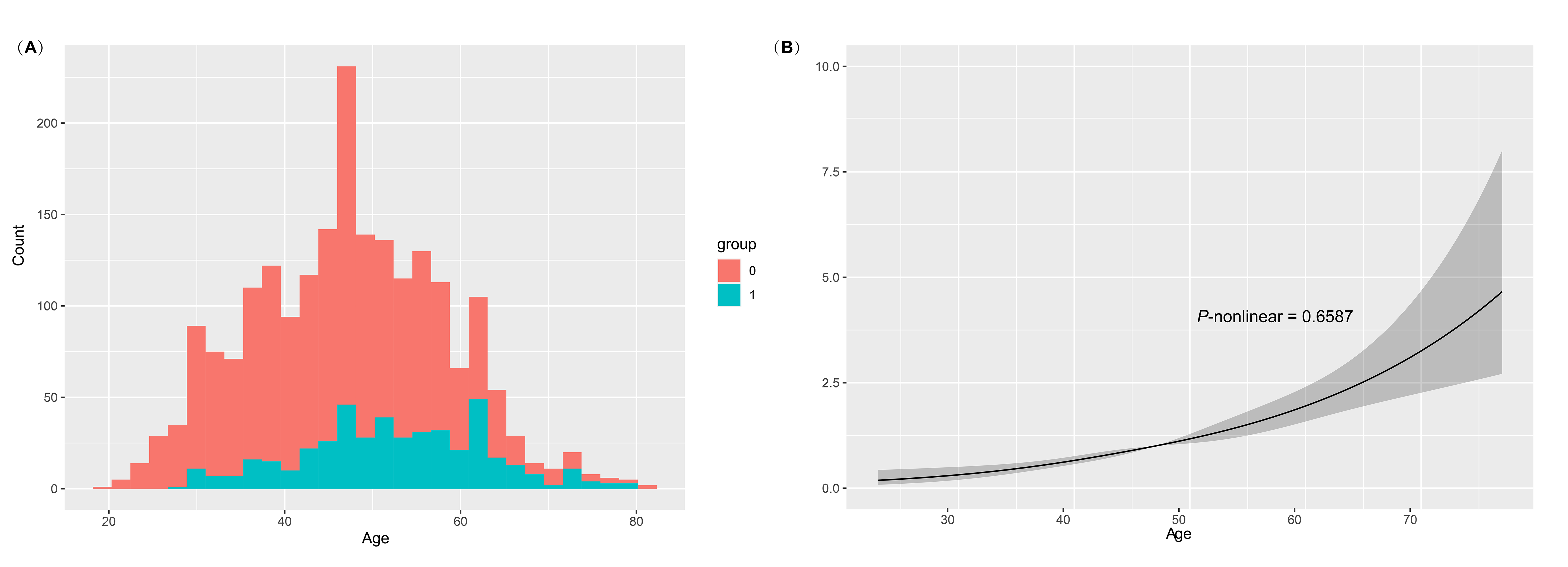

Supplement: Supplementary file 5 — Additional file 5: Figure S1. Age distribution of model development data and the association of age and ECC positivity (A-B). [file 12885_2021_8523_MOESM5_ESM.tif]

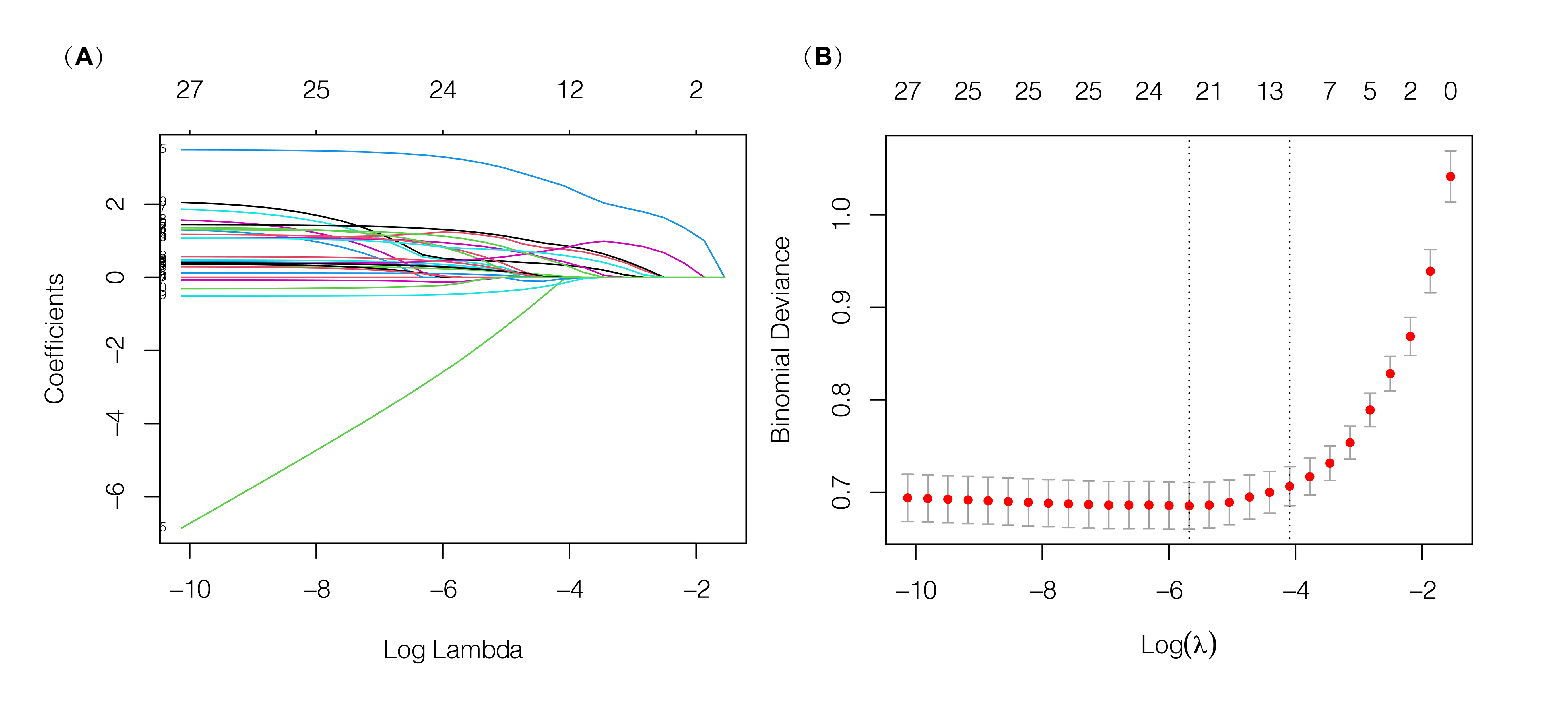

Supplement: Supplementary file 6 — Additional file 6: Figure S2. The progress of LASSO regression selecting the candidate variables and the penalty parameter λ. [file 12885_2021_8523_MOESM6_ESM.tif]
